# The Difficulties of the British Army Medical Officer

**Published:** 1898-09

**Authors:** 


### THE DIFFICULTIES OF THE BRITISH ARMY MEDICAL OFFICER.

A correspondent signing himself P. O., writing to the *Indian Lancet* for July 1st on the future of the army medical staff, says: The concession of military titles is an item of their claim; but they have other needs than this. Lord Landsdowne can not really think that he has done all that is necessary when he abolishes the designation which connects medical men with the profession to which they have the honor to belong. The rates of pay and allowances are none too liberal —a veterinary lieutenant is more highly paid than a surgeon lieutenant—while the treatment to which medical officers are subjected in regard to the changes of station is little less than scandalous. The tour of service now is running a short two years at home as against a long five years abroad; and what is worse, during the home tour there is no fixed tenure. A surgeon may be in Ireland one week, in Scotland the next, and perhaps in the south of England before the month is out. If he should be married, as he generally is, and takes and furnishes a house, as he often weakly does, it almost surely follows that he is ordered off somewhere else at a few days' notice. All this is bad enough for him, but it may be even worse for his patients. The other day in a garrison town an officer had measles in his family. The family were attended by five different medical officers during six successive days, and if the children got well it was because the captain who was their father—albeit he was a captain without being a medical man—doctored them himself. What medical officers most need is considerate and reasonable treatment from headquarters and considerate and reasonable treatment from those with whom they live in daily association.—*New York Medical Journal.*

